# Hospitalized Children With Familial Hypercholesterolemia and COVID-19: A Case for Preventive Anticoagulation

**DOI:** 10.3389/fcvm.2021.657719

**Published:** 2021-04-20

**Authors:** Alpo Vuorio, Frederick Raal, Petri T. Kovanen

**Affiliations:** ^1^Mehiläinen Airport Health Centre, Vantaa, Finland; ^2^Department of Forensic Medicine, University of Helsinki, Helsinki, Finland; ^3^Faculty of Health Sciences, University of Witwatersrand, Johannesburg, South Africa; ^4^Atherosclerosis Laboratory, Wihuri Research Institute, Helsinki, Finland

**Keywords:** familial hypercholesterolemia, COVID-19, D-dimer, anticoagulation, endotheliitis

## Introduction

Heterozygous familial hypercholesterolemia (HeFH) affects about one in 200 to 250 persons or over 30 million people worldwide, of whom about 20–25% are children and adolescents (1, 2). In those with HeFH, the level of serum low-density lipoprotein cholesterol (LDL-C) is elevated about two-fold from birth (3). If left untreated, the severe hypercholesterolemia causes pre-mature atherosclerosis. The standard treatment in HeFH children is statin therapy, which should start when the child is between 8 and 12 years of age (4). Homozygous familial hypercholesterolemia (HoFH) is the severe form of familial hypercholesterolemia (FH) affecting approximately 1 in 300,000 persons worldwide and causing four- to five-fold elevated levels of serum LDL-C (5). Despite the availability of multiple lipid-lowering therapies most HoFH patients do not achieve sufficiently low LDL-C levels, and accordingly are at high risk of symptomatic atherosclerotic cardiovascular disease already in childhood (6). In fact, there have been several case reports of sudden cardiac death due to fatal myocardial infarction in children with HoFH before the age of 10 years (7). Of note, the majority of the clinical studies performed on FH patients have included only the much more common form of FH, i.e., HeFH. Accordingly, when we refer to mere “FH,” we refer to studies with HeFH patients, unless specified otherwise.

## Endothelial Dysfunction in Familial Hypercholesterolemia

The significantly elevated serum LDL-C causes endothelial dysfunction already in young children with FH (8, 9). Additionally, many FH patients have raised serum levels of lipoprotein(a) [Lp(a)] (10). Thus, endothelial function in FH children can be severely compromised when both LDL-C and Lp(a) levels are increased (8). Moreover, compared with unaffected controls, FH children display a proinflammatory and prothrombotic phenotype which is associated with vascular dysfunction (11). Because Lp(a) is circulating in the blood, both proinflammatory and antifibrinolytic (i.e., prothrombotic) effects may extend from the macrovascular to the microvascular level, so affecting the entire circulatory system. Furthermore, because Lp(a) inhibits fibrinolysis, the risk of forming non-occluding or occluding thrombi is increased in FH children, in contrast to non-FH children with a primarily healthy endothelium (12).

## COVID-19–An Endothelial Disease

COVID-19 is considered to be an endothelial disease (13). Thus, the effect of this disease on vessel wall endothelial linings should particularly affect FH patients with COVID-19, in whom the already dysfunctional endothelium is acutely exposed to additional damaging insults caused by the excessive immunoinflammatory response of the host (i.e., the cytokine storm) and because the coronavirus can damage the endothelial cells also directly thereby leading to “endotheliitis” (13, 14). When exposed to inflammatory and infectious signals, the normally anticoagulant, antithrombotic, and profibrinolytic endothelial cells become activated and locally promote the activation of the coagulation cascade and thrombus formation. The pro-coagulant/pro-aggregatory, pro-inflammatory, vasoconstrictor, pro-oxidant, and barrier function-impairing properties of such damaged endothelium then critically contribute to the multiorgan failure characteristic of advanced stages of COVID-19.

## COVID-19 Is a Prothrombotic State

A recent autopsy study revealed that adult COVID-19 patients frequently have fibrin microthrombi in the heart without acute ischemic injury (15). The risk of developing such non-occluding or even occluding cardiac microthrombi is likely to be higher in children with FH. According to the results of a recent meta-analysis, among hospitalized adult patients with COVID-19, the prevalence of acute myocardial infarction was 3.3% (95% CI 0.3–8.5) (16). Therefore, the possibility that children with FH, particularly those with HoFH and COVID-19, are at increased risk of coronary thrombus formation and, despite their young age, may be at risk for an ischemic cardiac event (6).

Current data have demonstrated a COVID-19-induced prothrombotic state in children, as reflected by elevated D-dimer levels (17). This prothrombotic state can be further followed in the clinical setting by using a diagnostic disseminated intravascular coagulation (DIC) score, which has been established by The International Society on Thrombosis and Haemostasis (ISTH) (18, 19). The ISTH DIC score is taking into account several mechanisms related to the DIC syndrome which is characterized by widespread intravascular activation of coagulation. The pathophysiological mechanisms include, among others, cytokine-initiated inflammatory activation of coagulation and insufficient control of anticoagulant pathways, which together lead to endothelial dysfunction and microvascular thrombosis (19). The usefulness of the ISTH DIC score was shown in a retrospective large cohort study of 1,127 adult COVID-19 patients in Spain (20). In this study, the initial ISTH DIC score was significantly higher among the ultimately non-surviving patients.

Al-Ghafry et al. (21) recently published a case series of eight hospitalized COVID-19 pediatric patients, in which the coagulation profiles were determined. Six children had elevated D-dimer levels and required oxygen supplementation, and five children also required intensive care unit treatment. The authors carried out rotational thromboelastometry and found an increased blood clot firmness with a contribution from fibrinogen. Based on these laboratory findings, the children of whom the youngest were 8 years old received anticoagulation according to institutional adult anticoagulation guidelines, and no thromboembolic complications were observed in the treated children. Based on the above findings there is a potential need to expand and study the indication for prophylactic anticoagulation in hospitalized children with COVID-19 (22) to children with FH, provided there are no contraindications to anticoagulant therapy. Furthermore, in FH children, it is essential to continue effective statin therapy because statins not only improve endothelial function but also decrease serum D-dimer levels by about 15%, thus providing additional mild anticoagulation (23, 24). Moreover, because proprotein convertase subtilisin/kexin type 9 (PCSK9) inhibitors effectively lower serum LDL-C concentration, reduce the Lp(a) level by about 30%, and may also enhance the antiviral action of interferon in patients with hypercholesterolemia, the use of these inhibitors could be considered in hospitalized pediatric FH patients with COVID-19, particularly those with HoFH, if not already in use (25–27).

## Discussion

Results from controlled studies investigating the clinical effects of anticoagulation in hospitalized children with COVID-19 are lacking. Meanwhile, Loi et al. (22) have recommended that children with COVID-19 are eligible for anticoagulation. Based on the considerations presented here and on a recent expert consensus-based pediatric opinion (28), anticoagulant prophylaxis in children should be carried out (in the absence of any contraindications) by using low-dose low-molecular-weight heparin. Loi et al. also recommend that, in hospitalized children with COVID-19, it is important to trend the disseminated intravascular coagulation score with attention to the D-dimer level. Additionally, a pediatric risk assessment and consideration of prophylactic anticoagulation to prevent thrombosis should be performed at baseline and daily thereafter. When considering that FH is a prothrombotic condition by itself, the above recommendations would particularly apply to hospitalized FH children with COVID-19 (10, 29). This idea is supported by the above consensus-based clinical recommendation for anticoagulation in children hospitalized for COVID-19-related illnesses (28).

## Author Contributions

AV: writing the first draft. AV, FR, and PK: reviewing and editing to produce the final draft. All authors contributed to the article and approved the submitted version.

## Conflict of Interest

PK has received consultancy fees, lecture honoraria and/or travel fees from Amgen, Novartis, Raisio Group, and Sanofi. FR has received research grants, honoraria, or consulting fees for professional input and/or delivered lectures from Sanofi, Regeneron, Amgen, and Novartis. The remaining author declares that the research was conducted in the absence of any commercial or financial relationships that could be construed as a potential conflict of interest.
